# Clinical considerations in individuals with α_1_-antitrypsin PI*SZ genotype

**DOI:** 10.1183/13993003.02410-2019

**Published:** 2020-06-18

**Authors:** Gerard N. McElvaney, Robert A. Sandhaus, Marc Miravitlles, Gerard M. Turino, Niels Seersholm, Marion Wencker, Robert A. Stockley

**Affiliations:** 1 Dept of Respiratory Medicine, Beaumont Hospital, Royal College of Surgeons in Ireland, Dublin, Ireland; 2 Division of Pulmonary, Critical Care and Sleep Medicine, National Jewish Health, Denver, CO, USA; 3 Pneumology Dept, Vall d'Hebron University Hospital/Vall d'Hebron Research Institute (VHIR), CIBER de Enfermedades Respiratorias (CIBERES), Barcelona, Spain; 4 Dept of Medicine, Mt Sinai-St Luke's-Roosevelt Hospital, New York, NY, USA; 5 Dept of Respiratory Medicine, Gentofte Hospital, Hellerup, Denmark; 6 Conresp, Loerzweiler, Germany; 7 Lung Investigation Unit, University Hospitals Birmingham NHS Foundation Trust, Birmingham, UK

## Abstract

α_1_-Antitrypsin deficiency (AATD), characterised by reduced levels or functionality of α_1_-antitrypsin (AAT), is a significantly underdiagnosed genetic condition that predisposes individuals to lung and liver disease. Most of the available data on AATD are based on the most common, severe deficiency genotype (PI*ZZ); therefore, treatment and monitoring requirements for individuals with the PI*SZ genotype, which is associated with a less severe AATD, are not as clear. Recent genetic data suggest the PI*SZ genotype may be significantly more prevalent than currently thought, due in part to less frequent identification in the clinic and less frequent reporting in registries. Intravenous AAT therapy, the only specific treatment for patients with AATD, has been shown to slow disease progression in PI*ZZ individuals; however, there is no specific evidence for AAT therapy in PI*SZ individuals, and it remains unclear whether AAT therapy should be considered in these patients. This narrative review evaluates the available data on the PI*SZ genotype, including genetic prevalence, the age of diagnosis and development of respiratory symptoms compared with PI*ZZ individuals, and the impact of factors such as index *versus* non-index identification and smoking history. In addition, the relevance of the putative 11 µM “protective threshold” for AAT therapy and the risk of liver disease in PI*SZ individuals is explored. The purpose of this review is to identify open research questions in this area, with the aim of optimising the future identification and management of PI*SZ individuals.

## Abstract 2

Individuals with α_1_-antitrypsin (AAT) PI*SZ genotype appear to have an increased risk for lung and liver disease, although definitive evidence is lacking; smoking is a major risk factor for lung disease. The role of AAT therapy requires further study. http://bit.ly/2TxxFD0

## Introduction

α_1_-Antitrypsin deficiency (AATD) is an underdiagnosed orphan genetic condition that predisposes individuals to the development of lung and liver disease, and is characterised by reduced serum levels and/or functionality of α_1_-antitrypsin (AAT) [1]. An imbalance between protease and antiprotease activity may lead to accelerated lung tissue degradation, especially by neutrophil elastase, and progression to COPD with early-onset emphysema. In patients with severe AATD and emphysema, intravenous AAT therapy is the only available treatment that has demonstrated a disease-modifying effect and its early implementation is key to slowing disease progression [2, 3]. In addition to pulmonary complications, AATD is a risk factor for other serious illnesses, including the development of liver disease [4].

The most common deficiency alleles, which are associated with reduced serum levels of AAT, include PI*Z and PI*S [4]. AATD is most commonly associated with the Z variant; the mechanism leading to AATD is polymerisation of “Z” AAT proteins, which results in accumulation in the endoplasmic reticulum of hepatocytes, preventing secretion into plasma and hence a deficiency in circulating AAT [5]. In some individuals, the aggregation of abnormal protein in the endoplasmic reticulum can result in eventual progression to clinically overt liver disease, the severity of which correlates with the magnitude of accumulation [6].

Severe AATD is most commonly associated with homozygosity for the Z allele (PI*ZZ). In individuals with this genotype, extensive polymerisation of AAT within hepatocytes results in very low AAT plasma levels (<10 µM/<52 mg·dL^−1^) compared with the normal range (17–47 µM/102–254 mg·dL^−1^) [7], putting them at a high risk of developing emphysema. This typically presents clinically in the fourth and fifth decades of life, and is associated with an accelerated decline in pulmonary function and an increased frequency of exacerbations, which may be associated with disease progression [5]. As a result of codominant gene expression, individuals heterozygous for the Z allele (*i.e.* PI*MZ individuals) typically show AAT levels of 11–28 µM (62–151 mg·dL^−1^) [7], slightly below the normal range, and may, therefore, also be at some risk of developing lung and/or liver disease in combination with other risk factors [8]. However, the majority of data related to AATD are based on studies of patients with the PI*ZZ genotype and the clinical significance of compound heterozygotes has been a subject of great debate.

Individuals with the PI*SZ genotype usually have AAT levels between those observed in PI*ZZ and PI*MZ individuals, but are considered to have moderate-to-severe deficiency. Some evidence suggests that individuals with this genotype have an increased risk of developing COPD compared to PI*MM individuals [9]; however, this is not universally accepted or proven [10].

As a result, there is a lack of consensus on how PI*SZ individuals should be monitored and treated. In particular, in countries where augmentation therapy is available [11], patients with the PI*ZZ genotype and emphysema are prescribed AAT therapy and are monitored on at least an annual basis to assess presentation/progression of lung and liver manifestations [12]. However, for individuals with the PI*SZ genotype, the issue of disease risk and management requirements remains unclear. The aim of this review is to identify open research questions and raise awareness of the potential risk of emphysema/COPD associated with the PI*SZ genotype, in order to aid future research, and the diagnosis and management of affected individuals.

## Prevalence of the PI*SZ genotype

Classically, AATD is considered to be the PI*ZZ genotype; however, recent genetic prevalence data suggest the number of individuals worldwide with the PI*SZ genotype could be as much as 10 times more than those with the PI*ZZ genotype [13], and that almost half of the estimated 1.5 million people with the PI*SZ genotype live in Europe [13, 14]. Therefore, the number of individuals with the PI*SZ genotype may be higher than previously thought. This underestimation may be due in part to less frequent identification in the clinic and less frequent reporting in registries, in addition to the fact that not all individuals with the PI*SZ genotype develop clinically significant disease.

In national and international AATD registries, patients with the PI*SZ genotype generally constitute a smaller proportion of the total number of patients (1.0–21.3%), with the highest number of PI*SZ individuals reported by the Spanish registry (table 1) [15–25]. However, not all of these individuals have symptomatic disease (index cases), as some registries, *e.g.* the Danish and Irish targeted detection programmes, conduct screening of family members of diagnosed individuals specifically to identify non-index cases [24, 26]. Overall, the number of individuals with the PI*SZ genotype in registries may be impacted by the specific exclusion criteria in some registries (*e.g.* AAT levels >11 µM) [22] and several other factors, so that the number of symptomatic PI*SZ patients would be underestimated.

**TABLE 1:** Estimated prevalence of PI*SZ and PI*ZZ genotypes from registries

	**Subjects**	**Genotype**	
		PI*SZ	PI*ZZ
**ADAPT: The UK Registry** [15]	1203	135 (11.2)	930 (77.3)
**AlphaNet (USA)** [16, 17]	5523	504 (9.13)	3031 (54.9)
**Italian Registry** [18]	422	74 (17.5)	258 (61.1)
**German Registry** [20]	1066	109 (10.2)	820 (76.9)
**Spanish Registry** [21]	469^#^	100 (21.3)	348 (74.2)
**AIR** [19, 41]	4758	538 (11.3)	4031 (84.7)
**Alpha One Foundation (Ireland)** [25]	5520^¶^	275 (4.9)	305 (5.5)
**NHLBI** [22]	1021	10 (1.0)	993 (97.3)
**AOF-RNR** [23]	712	15 (2.1)	503 (70.7)

Data are presented as n or n (%). ADAPT: Antitrypsin Deficiency Assessment and Programme for Treatment; AIR: Alpha One International Registry; NHLBI: National Heart, Lung, and Blood Institute; AOF-RNR: Alpha One Foundation Research Network Registry. ^#^: only adult patients included; ^¶^: excluding PI*MM individuals.

While the presence of the PI*SZ genotype does not necessarily translate into clinically relevant phenotypic traits, data suggest that this genotype may be a “relatively” common risk factor in the general population for lung and/or liver disease based on predicted genetic prevalence. As with individuals with the PI*ZZ genotype, the severity of symptoms is highly variable and neither AAT levels or disease phenotype are sufficient to identify which patients have the SZ genotype or will develop lung and/or liver disease. The development and severity of disease likely depends on multiple factors independent of genetics, and the ratio of lung to liver disease in individuals with the PI*SZ genotype is unclear; this is discussed further in the liver disease section. Additional factors that increase disease risk in PI*SZ patients may help in the identification of symptomatic patients and inform management requirements. Knowledge of prevalence may lead to increased identification of affected individuals, a better understanding of “risk” and potential implementation of preventive measures, such as aggressive smoking cessation.

Research questions: What is the true prevalence of the PI*SZ genotype worldwide? What is the true prevalence of lung disease in individuals with the PI*SZ genotype?

## The PI*SZ genotype: the significance of AAT levels

The “S” mutation is known to have less impact on the circulating AAT protein than the “Z” mutation, with the S allele resulting in AAT secretion of up to 60% compared to the normal “M” variant. By comparison, the Z allele typically results in secreted levels that are ∼15% of normal [4, 27]. This difference is attributed to the lower propensity of the S than the Z protein to polymerise [28]; however, the S variant is also capable of forming heteropolymers with the Z variant [29]. As a result, individuals with the PI*SZ genotype generally have markedly reduced AAT levels, although higher than those observed in individuals with the PI*ZZ genotype [4, 30–32].

AAT levels associated with the PI*SZ genotype became part of the basis for defining the putative 11-µM “protective threshold” for AATD risk and therapeutic targeting because of a generally perceived low risk of disease. As PI*SZ patients typically have AAT levels 25–40% of normal, with the presence of emphysema in some patients, it was originally theorised that the protective threshold should be ∼35% of normal levels [33]. Since then, the American Thoracic Society/European Respiratory Society (ERS) 2003 statement, with original citations dating back to the 1980s, is often used as the reference guide, although more recent publications using modern assays have updated the reference ranges for each genotype. Due to its theoretical nature, the current 11-µM threshold (≈57.2 mg·dL^−1^ by modern assays, or 80 mg·dL^−1^ if measured by the now obsolete radial immunodiffusion method [4, 34]) remains controversial, and now may be used less in clinical decision-making processes in some centres. However, an *in vitro* quantum proteolysis study did support the validity of a threshold of ∼11 µM (≈57.2 mg·dL^−1^), as a nonlinear relationship was found between AAT concentration and the magnitude of quantum proteolytic events, with a dramatic increase observed at AAT concentrations below ∼11 µM (≈57.2 mg·dL^−1^) [35]. Furthermore, using this threshold as a guide and raising AAT levels above this level with *i.v.* AAT therapy has been shown to be clinically effective in PI*ZZ individuals [36].

These data suggest that most PI*SZ individuals would not benefit from augmentation therapy. However, importantly, serum levels of AAT vary significantly in individuals with the PI*SZ genotype, and reported levels differ between studies (table 2) [4, 30, 31]. Recent data showed that 15.4% of individuals with the PI*SZ genotype had AAT levels below a cut-off of 50 mg·dL^−1^ (≈9.6 µM) compared with 96.9% of PI*ZZ individuals [31]. However, it is important to note that AAT is an acute-phase reactant, and as for C-reactive protein (CRP), levels of AAT can be transiently elevated by trauma, inflammation and hormonal changes [30, 37]. Therefore, elevated CRP levels are closely related to increases in AAT levels in PI*SZ individuals in a nonlinear manner, potentially masking their disease risk [37]. It is therefore advisable that simultaneous determination of CRP and AAT levels be conducted in PI*SZ individuals [37]. It should also be noted that patients with non-AATD-related COPD have higher levels of inflammation as a feature of the disease and thus, patients with the PI*SZ genotype and COPD may have higher baseline CRP, and hence AAT levels, which also correlate with the severity of COPD [38].

**TABLE 2:** Serum α_1_-antitrypsin (AAT) level ranges according to genotype

	**Genotype**			
	PI*MM	PI*MZ	PI*SZ	PI*ZZ
**ATS/ERS guidelines (2003)** [4]				
µM	20.0–48.0	17.0–33.0	8.0–16.0	2.5–7.0
mg·dL^−1^	150–350	90–210	75–120	20–45
**Ferrarotti*et al*. (2012)** [30]				
µM	20.2–31.5	12.7–19.2	9.4–12.7	NA
mg·dL^−1^	105–164	66–100	49–66	NA
**Bornhorst*et al*. (2013)** [31]				
µM	19–47	11–28	7–23	≤5–10
mg·dL^−1^	102–254	62–151	38–108	≤29–52

ATS: American Thoracic Society; ERS: European Respiratory Society; NA: not available.

**FIGURE 1:**
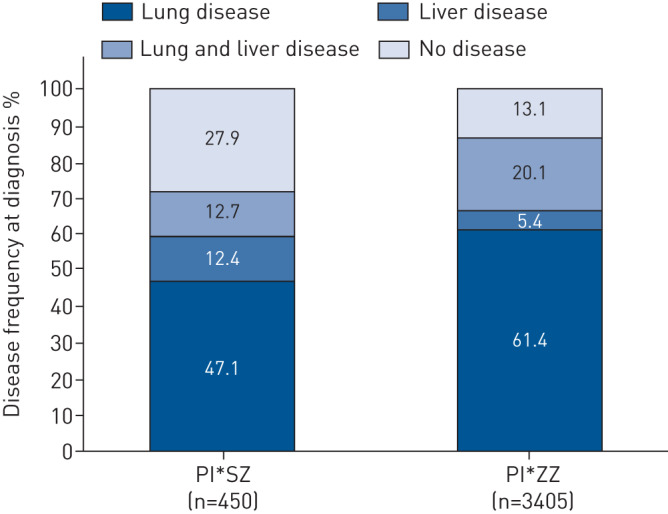
Frequency of lung and/or liver disease at diagnosis according to genotype in the Alpha One International Registry (AIR) (unpublished observations).

Overall, little is known about the functional capacity of many of the rarer AAT genotypes; however, prevailing serum AAT levels relate to disease severity, and PI*SZ individuals have a lower risk of emphysema, and a better survival than PI*ZZ patients [32, 39, 40]. This is confirmed by data from the Alpha One International Registry (AIR) demonstrating that the PI*SZ genotype is associated with a lower frequency of lung disease and better health than the PI*ZZ genotype (figure 1) [41]. Nonetheless, some bias may have been introduced by preferentially including individuals with AAT levels below the putative 11-µM (≈57.2 mg·dL^−1^) threshold, which may reflect those known to be or assumed to be at an increased risk of lung disease [4]. This hypothesis would be dependent on referral patterns or the tendency to measure AAT levels first and only assess phenotype/genotype if the level was found to be very low.

Research question: How do AAT levels in individuals with the PI*SZ genotype relate to disease risk, and is the subgroup of patients with lower AAT levels (<11 µM (≈57.2 mg·dL^−1^)) at an increased risk of lung disease?

## Influence of smoking

Smoking is a key risk factor for the development of lung disease in patients with AATD, and disease progression and survival are both significantly worse in smokers than never-smokers [4, 42]. Recent studies have shown that identified PI*SZ individuals are more likely to have a history of tobacco smoking, and generally worse health than PI*ZZ individuals, and demonstrate worse behaviours associated with poor health (such as lack of exercise) [16, 17]. However, there are conflicting reports as to the individual impact of smoking on the development of lung disease in PI*SZ individuals.

Some studies have suggested that PI*SZ never-smokers, such as those in the Swedish neonatal screening cohort, have normal lung function aged 37–40 years [43, 44]. By comparison, it is probable that a history of smoking in PI*SZ individuals contributes to an increased risk of developing lung disease and hence a higher rate of mortality than PI*SZ never-smokers [26, 45]. However, it is important to note that in a Danish registry, over one-third of PI*SZ index cases, ascertained due to respiratory symptoms, were never-smokers (10 out of 28 patients), suggesting that other factors contribute to the development of respiratory disease at least in some PI*SZ individuals [26]. Importantly, despite having a higher average smoking consumption, PI*SZ individuals usually have better preserved lung function compared with matched PI*ZZ individuals [46]. The UK AATD registry reported that PI*SZ individuals appear less susceptible to the harmful effects of cigarette smoking than PI*ZZ patients [47], potentially indicating a dose–response curve interacting between AAT level and smoking status. The impact of smoking has been found to be greater at the beginning of the habit, with a faster rate of decline in lung function observed with the first 20 pack-years for both PI*SZ and PI*ZZ genotypes [46].

The Irish AATD registry previously established that in PI*MZ individuals, only those who smoke have an increased risk for developing COPD [8], and it is currently unclear whether a similar relationship exists for PI*SZ individuals, although the lower AAT level in such individuals suggests that it should. A study of PI*SZ individuals in the Irish registry aims to answer this question and is expected to be published in the near future. It is concerning that PI*SZ individuals appear to exhibit higher rates and higher pack-years of smoking than PI*ZZ individuals. Smoking cessation remains the most important intervention in all patients with AATD, and preventing smoking initiation should be strongly encouraged; however, this requires widespread screening to identify such subjects prior to the uptake of the smoking habit.

Exposure to occupational and environmental pollutants that cause respiratory irritation (*e.g.* gases/fumes used in the agricultural industry or long-term ozone exposure) are known to be independent risk factors for lung function impairment in PI*ZZ and PI*MZ individuals, and should be avoided where possible to help maintain good lung health [1, 48, 49]. Currently, there are no published data pertaining to the effects of occupational and environmental pollutants in PI*SZ individuals, but it is reasonable to assume that at least some patients may be as vulnerable as PI*ZZ patients [4].

Research questions: What is the impact of smoking on the development of lung disease in PI*SZ individuals and is there an increased risk of lung disease in never-smokers? How does smoking cessation affect the course of lung disease in PI*SZ individuals? Are there environmental risk factors that contribute to disease progression and morbidity?

## Age of onset and index *versus* non-index cases

PI*SZ individuals generally develop respiratory symptoms at a later age than individuals with more severe AATD (*i.e.* PI*ZZ, PI*Z null and PI*null null individuals), potentially reflecting the higher average AAT concentrations in PI*SZ individuals [26, 46] and hence a slower disease evolution. The mean age at diagnosis for individuals with the PI*SZ genotype ranges from 45–46 years for non-index cases identified through family screening to 51–55 years for index cases [26, 50]. Pulmonary emphysema is often present in PI*SZ index cases, particularly if they are current or ex-smokers [51]. It has also previously been reported that index PI*SZ patients have reduced percentage of predicted forced expiratory volume in 1 s and survival compared with the general population [26]. Importantly, data from the Danish registry suggest that non-index PI*SZ cases have better survival than index cases, with age at death 65–87 years and 45–74 years, respectively [26]. Nonetheless, the main causes of death in both index and non-index cases were respiratory related (pulmonary emphysema, pulmonary fibrosis and pneumonia).

Overall, the prognosis of PI*SZ individuals may be strongly influenced by their age at diagnosis, smoking history and whether they are identified as index or non-index cases. This highlights the value of familial screening [12], which enables the identification of individuals with the PI*SZ genotype at a younger age, facilitating early healthcare advice.

Research questions: What is the prognosis for individuals with the PI*SZ genotype and does early diagnosis through familial screening lead to lifestyle changes that influence the development and progression of lung disease? What factors, in addition to age and smoking, may contribute to individuals being identified as an AATD index case? What is the best strategy for early diagnosis of SZ individuals as index cases?

## Emphysema distribution

Following the development of respiratory symptoms in patients with AATD, the presence/extent of emphysema should be assessed, as this impacts treatment and potentially prognosis and monitoring decisions. Computed tomography (CT) has been widely used to characterise emphysema distribution in PI*ZZ patients; however, the presentation of emphysema in PI*SZ patients has been less well defined.

Most of the available data regarding the extent and distribution of emphysema in PI*SZ patients have been provided by the UK Antitrypsin Deficiency Assessment and Programme for Treatment (ADAPT) registry. These data suggest that emphysema may be less prevalent in PI*SZ than PI*ZZ individuals: fewer than half of the individuals with the PI*SZ genotype had CT scan evidence of emphysema (35%) compared with those with the PI*ZZ genotype (86%) [52]. It has been suggested that emphysema distribution in PI*SZ patients has both characteristics of non-AATD COPD and PI*ZZ-related emphysema. Classically, emphysema in PI*ZZ individuals has been described as panacinar disease affecting predominantly the basal region, compared with the mainly centrilobular and apical distribution seen in non-AATD COPD [53, 54]. Although emphysema distribution is primarily observed to be homogenous in AATD, a significant number of patients present with heterogeneous/apical emphysema, *i.e.* more similar to non-AATD COPD [55]. Data from the UK registry suggest that individuals with the PI*SZ genotype have a higher proportion of apical emphysema than PI*ZZ individuals [32, 52] and the lower prevalence of emphysema may suggest that these patients have less parenchymal destruction than PI*ZZ patients [52]. Alternatively, it has been proposed that this could be a reflection of the early-phase of disease progression in some patients. However, the proportions of PI*SZ patients in the UK registry with panacinar only and centrilobular only emphysema were indistinguishable from those of PI*ZZ individuals [52]. This may suggest that a similar pathological process is taking place in both genotypes, but is distinct from purely smoking-related emphysema. Additionally, this may suggest that the use of AAT therapy could be justified for some PI*SZ patients with a phenotype of emphysema suggestive of a systemic cause, *i.e.* in patients with panacinar emphysema (currently, AAT therapy is not indicated for non-AATD COPD). However, clinically evident emphysema does not appear to develop in all patients with the PI*SZ genotype, suggesting that implementation of AAT therapy would generally have less justification in these patients.

Overall, the type of emphysema in PI*SZ patients appears to be intermediate between PI*ZZ patients and non-AATD COPD patients. It is important to note that the above data are from a small number of patients from one country and further studies with larger sample sizes in PI*SZ individuals are required to consolidate/generalise these findings. The increased sensitivity and usage of CT scans may prove crucial in the future for identifying PI*SZ patients with mild and asymptomatic emphysema.

Research questions: Is the morphology of emphysema associated with the PI*SZ genotype distinct from that of the PI*ZZ genotype, and if so, what are the clinical implications? Does emphysema distribution differ in early- and late-stage disease in individuals with the PI*SZ genotype?

## Liver diseases and other less frequent manifestations

Cirrhosis has been observed in both adult and paediatric patients with the PI*ZZ genotype; it is estimated that 10% of adults presenting with AATD-related liver disease have liver cirrhosis and will require transplantation [56]. Age and sex, in addition to preventable risk factors including body mass index, hepatitis B and C infection and alcohol consumption may predispose patients to liver disease [56, 57]. Although the prevalence and putative mechanism of the development of liver disease in PI*SZ individuals is less well understood, patients can develop similar liver biopsy abnormalities to those observed in PI*ZZ patients [4, 34]. In adults, significant liver fibrosis (≥F2; mild–moderate fibrosis) is commonly observed in both PI*SZ and PI*ZZ individuals, but severe liver fibrosis (≥F3; bridging fibrosis that has spread to different areas of the liver) rarely develops in PI*SZ patients [58]. While liver disease in PI*SZ individuals may be less prevalent and less clinically severe than in PI*ZZ individuals, it could be subclinical and remain undetected unless specifically sought. Enhanced liver fibrosis tests may identify those who are at risk of liver fibrosis and potentially developing clinically important cirrhosis [59].

The development of AATD-related liver disease in children differs from that of adults: neonates with the PI*ZZ genotype occasionally develop a fulminating hepatitis syndrome, with some reports of individuals developing cirrhosis who then require liver transplantation [56, 60]. Compared with patients with non-AATD-related neonatal hepatitis syndrome, it has been reported that the condition presents earlier and symptoms persist for longer in individuals with the PI*ZZ genotype [61]. However, previous studies have suggested that clinical evidence of liver disease and/or abnormal liver enzymes are rarely observed in PI*SZ infants, and in those with enzyme abnormalities, levels normalise within the first year of life [62, 63]. More recently, a retrospective study in children diagnosed with the PI*SZ genotype identified symptoms of liver disease, such as prolonged jaundice, abdominal swelling and loss of appetite, at a median age of 3 months. However, asymptomatic siblings had no clinical signs of liver disease, highlighting the variability of liver involvement in PI*SZ individuals [64].

Since no specific pharmacological treatment for AATD-related liver disease is currently available, liver transplant is the only treatment option for decompensated cirrhosis and liver failure. A study of AATD-related liver transplantation in 50 PI*ZZ and 23 PI*SZ cases [65], reported that PI*SZ patients were older at the time of transplant (53 *versus* 47 years), and a greater proportion of patients had an underlying concomitant cause of liver disease (such as hepatitis C virus or alcohol-induced liver damage) compared with PI*ZZ patients (43.5% *versus* 8%). In addition, despite age being a modifying factor, PI*SZ patients had better survival than PI*ZZ patients (although not statistically significant) [65]. Compared with the PI*MZ genotype, there is evidence to suggest that the PI*SZ genotype confers greater risk of liver disease [65] which may be due to higher levels of heteropolymers as *in vivo* studies suggest that the M and S proteins colocalise with the Z variant within liver cells, but with greater accumulation of ZZ complexes than the SZ and MZ complex (ZZ>SZ>MZ) [66]; however, further research should be undertaken to confirm this. Liver comorbidities such as hepatitis B and C have given rise to the “second hit” theory, whereby the associated liver pathology could be worsened in the presence of AATD [67]. Preventative measures such as vaccination and alcohol avoidance/moderation may help to minimise these liver-associated complications later in life.

Overall, the risk of liver disease in PI*SZ individuals is much lower than that of PI*ZZ individuals; however, it remains an important aspect of the disease risk associated with PI*SZ genotype. It is notable that in the AIR, the prevalence of liver disease in PI*SZ individuals was similar to that of the PI*ZZ individuals, with a higher frequency of liver disease observed in isolation (without lung disease) in PI*SZ than PI*ZZ individuals (figure 1). However, this may be an overestimation due to ascertainment bias, *i.e.* a higher proportion of index liver cases in the PI*SZ group, or just the higher prevalence of the PI*SZ genotype in general, and patients identified for specialist liver clinics.

Other extrapulmonary manifestations of AATD that have predominantly been linked to the PI*ZZ genotype, but have also been observed in individuals with the PI*SZ genotype include panniculitis and ANCA-negative vasculitis [68, 69]. However, there is a lack of literature on these aspects and further research is required.

Research question: What is the prevalence of liver abnormalities in individuals with the PI*SZ genotype and what are the risk factors for the progression to clinically significant liver disease? Is the risk of liver disease higher in the SZ genotype compared with the MZ genotype?

## Implications and clinical considerations

### Identifying PI*SZ individuals

National and international guidelines recommend that all patients with COPD, nonreversible asthma and unexplained liver disease should be tested for AATD [4, 12, 70, 71]. Despite these recommendations, identification of AATD remains low due to a continued lack of disease awareness and poor adherence to testing recommendations [72, 73]. It has been suggested that this reflects the erroneous perception of high costs associated with testing [12, 72]. Measures such as the cost-effective detection programme set up by the Spanish registry of patients with AATD may help to alleviate this concern and improve diagnosis [72]. In addition, guidelines strongly advocate the use of familial testing [12, 71], and given the less-severe disease observed in PI*SZ individuals, familial testing may be especially useful in identifying these individuals who may remain asymptomatic until later life, as demonstrated by the Irish registry [24].

Research questions: How can the diagnosis of individuals with the PI*SZ genotype be improved and what is the significance of early diagnosis? How can early diagnosis facilitate a better understanding of the implications of the PI*SZ genotype?

### Implementing AAT therapy in PI*SZ patients

AAT augmentation therapy is less frequently used in PI*SZ patients than PI*ZZ patients [46], which is consistent with evidence of a reduced risk of lung disease in PI*SZ individuals (as summarised earlier). The US Alpha 1 Foundation guidelines [71] do not preclude the use of AAT therapy in these patients, whereas the recent ERS statement on AATD [12] indicates that there is no current evidence to support efficacy of augmentation therapy in any PI*SZ individual. Currently, adequate clinical studies of the use of AAT therapy in PI*SZ individuals are lacking, which partly explains this disparity. Sufficiently powered randomised controlled trials are required to verify a treatment effect of AAT therapy in PI*SZ patients, using a comparable primary end-point to previous clinical trials in PI*ZZ patients, *i.e.* CT lung density. Additionally, it would be critical to compare any treatment effect in PI*SZ individuals and whether the baseline blood AAT levels are above and below the putative 11-µM threshold. At present, whether AAT levels are above or below the 11-µM protective threshold should not in itself guide treatment decisions, and in the absence of evidence, this should possibly be based on the accumulation of other risk factors including younger age of symptom onset, smoking status, confirmed emphysema on CT and its progression despite smoking cessation.

Research question: Is AAT augmentation therapy indicated and effective in slowing emphysema progression in individuals with the PI*SZ genotype?

## Conclusions

This review highlights several open research questions requiring further investigation in patients with the PI*SZ AAT genotype. The prevalence of the PI*SZ genotype is greater than that of the PI*ZZ genotype (especially in Mediterranean countries), and some PI*SZ individuals are probably at a low, albeit increased, risk of developing lung and liver disease compared to individuals with the normal genotype. Additional research is needed to clarify the risk of COPD/emphysema and identify PI*SZ individuals who may be at the highest risk of developing lung disease. Gaining further insight regarding the benefits of AAT therapy in PI*SZ patients who have low serum AAT levels (*e.g.* <11 µM) may help guide future treatment decisions in these individuals.

Further data are required on the influence of smoking (including current and ex-smokers) on disease development and progression in PI*SZ patients and particularly how this relates to index *versus* non-index status. Given the greater prevalence of the PI*SZ than the PI*ZZ genotype, there is potentially a significant proportion of patients who may benefit from effective antiproteinase therapy. In particular, the benefit of augmentation therapy should be explored through appropriate clinical trials, with priority given to patients whose clinical course mimics that of patients with the PI*ZZ genotype. Moreover, longitudinal registry and epidemiology data assessing index and non-index cases are required to identify genotype-specific abnormalities, risk factors, rate of progression and help inform therapeutic requirements. The new European Α1-Research Collaboration (EARCO) registry aims to recruit and observe a significant number of PI*SZ individuals to answer some of these questions [74]. Furthermore, the PI*SZ genotype appears to be a risk factor for liver abnormalities and additional studies of liver involvement and progression in PI*SZ individuals may provide a clearer picture as to how they should be monitored and counselled.

Although guidance for the management of PI*SZ patients would be beneficial to clinicians, the present article indicates that there is currently a lack of evidence for the firm basis of such guidance other than to provide appropriate management of the disease comorbidities. Future research initiatives should focus on the knowledge gaps raised in this article.

## Shareable PDF

10.1183/13993003.02410-2019.Shareable1 This one-page PDF can be shared freely online.
Shareable PDF ERJ-02410-2019.Shareable

## Supplementary Material

ERJ-02410-2019.Shareable.pdf
