# Supplementary material for: Clinical considerations in individuals with α1-antitrypsin PI*SZ genotype
Source: Eur Respir J. 2020 Jun 18;55(6):1902410. doi: 10.1183/13993003.02410-2019 (PMC7301289; doi:10.1183/13993003.02410-2019)
Supplement: Supplementary file 1 [file ERJ-02410-2019.Shareable.pdf]

SHAREABLE PDF

Clinical considerations in individuals with α_1-antitrypsin PI*SZ genotype

Gerard N. McElvaney[1], Robert A. Sandhaus[2], Marc Miravitlles[3], Gerard M. Turino[4], Niels Seersholm[5], Marion Wencker[6] and Robert A. Stockley[7]

Affiliations: [1]Dept of Respiratory Medicine, Beaumont Hospital, Royal College of Surgeons in Ireland, Dublin, Ireland. [2]Division of Pulmonary, Critical Care and Sleep Medicine, National Jewish Health, Denver, CO, USA. [3]Pneumology Dept, Vall d'Hebron University Hospital/Vall d'Hebron Research Institute (VHIR), CIBER de Enfermedades Respiratorias (CIBERES), Barcelona, Spain. [4]Dept of Medicine, Mt Sinai-St Luke's-Roosevelt Hospital, New York, NY, USA. [5]Dept of Respiratory Medicine, Gentofte Hospital, Hellerup, Denmark. [6]Conresp, Loerzweiler, Germany. [7]Lung Investigation Unit, University Hospitals Birmingham NHS Foundation Trust, Birmingham, UK.

Correspondence: Robert A. Stockley, Lung Investigation Unit, University Hospitals Birmingham NHS Foundation Trust, Queen Elizabeth Hospital Birmingham, Edgbaston, Birmingham, UK. E-mail: rob.stockley@uhb.nhs.uk

@ERSpublications
Individuals with α_1-antitrypsin (AAT) PI*SZ genotype appear to have an increased risk for lung and liver disease, although definitive evidence is lacking; smoking is a major risk factor for lung disease. The role of AAT therapy requires further study. http://bit.ly/2TxxFD0

Cite this article as: McElvaney GN, Sandhaus RA, Miravitlles M, *et al.* Clinical considerations in individuals with α_1-antitrypsin PI*SZ genotype. *Eur Respir J* 2020; 55: 1902410 [https://doi.org/10.1183/13993003.02410-2019].

This single-page version can be shared freely online.

ABSTRACT α_1-Antitrypsin deficiency (AATD), characterised by reduced levels or functionality of α_1-antitrypsin (AAT), is a significantly underdiagnosed genetic condition that predisposes individuals to lung and liver disease. Most of the available data on AATD are based on the most common, severe deficiency genotype (PI*ZZ); therefore, treatment and monitoring requirements for individuals with the PI*SZ genotype, which is associated with a less severe AATD, are not as clear. Recent genetic data suggest the PI*SZ genotype may be significantly more prevalent than currently thought, due in part to less frequent identification in the clinic and less frequent reporting in registries. Intravenous AAT therapy, the only specific treatment for patients with AATD, has been shown to slow disease progression in PI*ZZ individuals; however, there is no specific evidence for AAT therapy in PI*SZ individuals, and it remains unclear whether AAT therapy should be considered in these patients. This narrative review evaluates the available data on the PI*SZ genotype, including genetic prevalence, the age of diagnosis and development of respiratory symptoms compared with PI*ZZ individuals, and the impact of factors such as index *versus* non-index identification and smoking history. In addition, the relevance of the putative 11 µM "protective threshold" for AAT therapy and the risk of liver disease in PI*SZ individuals is explored. The purpose of this review is to identify open research questions in this area, with the aim of optimising the future identification and management of PI*SZ individuals.

Copyright ©ERS 2020. This version is distributed under the terms of the Creative Commons Attribution Licence 4.0.
